# Open questions: What is there left for cell biologists to do?

**DOI:** 10.1186/1741-7007-11-16

**Published:** 2013-02-27

**Authors:** Sean Munro

**Affiliations:** 1 Division of Cell Biology, MRC Laboratory of Molecular Biology, Hills Road, Cambridge CB2 0QH, UK

## 

There is little risk of cell biologists' getting bored in the 21^st ^century, but it is worth considering a few of the questions they might hope to have solved by 2100, if not before.

## Fundamental features of cells

Despite all the efforts of the 20^th ^century there are many areas of basic cell biology that remain mysterious. Some of these questions, such as how secreted proteins get across the Golgi, or the source of the membranes of autophagosomes, have been debated for so long that a further century might not be enough to reach agreement. Other phenomena such as lipid rafts, exosomes, or unconventional secretion seem to have divided the protagonists into camps of believers and skeptics who consider each other's views too eccentric to even engage. Other questions, however, have started to receive attention so recently that there may not yet be sufficient entrenched views to impede progress. These include the question of how organelles and cytoskeletal structures, and indeed the cell itself, maintain constant size and shape. In addition, the extent of non-vesicular transport of lipids between organelles has only recently been appreciated, and exciting recent work has revealed the importance of membrane contact sites [1], and also suggested that the transport of cholesterol to the plasma membrane is mediated by oxysterol binding proteins via a counter-current of phosphoinositides [2], although perhaps inevitably an opposing view has formed that oxysterol binding proteins do not actually perform cholesterol transport but instead activate an as yet unidentified transport machinery [3].

## Variations on a theme of Henrietta Lacks

Much of the work of the molecular era has concentrated on readily tractable cells, notably immortalized mammalian tissue culture cells, or powerful genetic systems such as yeast. Although many important general principles have emerged from these studies, they represent only a tiny fraction of the rich diversity of cell types that populate the protozoal world, or make complex multicellular creatures from oak trees to chimpanzees. One key challenge will be to understand how the basic machinery of organelles and cytoskeletal systems that all cells share is then regulated and enhanced to achieve this astonishing diversity.

## What cells should we care about?

It is unlikely that the planet's tax payers will be willing to pay for enough cell biologists to investigate every last intriguing invertebrate or bizarre bikont, and thus future work is likely to focus on particular key cells types, especially those found in tax payers themselves. Much of our body is made up of sheets of polarized epithelial and endothelial cells, whose shapes form tissues, and whose polarity allows the regulation of our internal fluids. How these cells are polarized is beginning to be understood, but there is still much to be learned about how proteins are directed to the different sides of these cells, and how their cytoskeletons are regulated to direct the changes in cell shape that form and maintain tissues.

An equally challenging and crucial question concerns the formation of the neurons and glia that are converting this text into your consciousness. We have a good, if not complete, understanding of how synapses work, but understand little of how membrane traffic and the cytoskeleton work together to establish and maintain the extraordinary cellular architecture of the brain.

## Life without growth

Another feature of the cells that most cell biologists study is that you find you have more of them when you return to the lab in the morning. This is of course very useful, but it has meant that relatively little work has been done on the cell biology of post-mitotic or quiescent cells. Such cells form the majority of our tissues, and in addition to their cell-type-specific features, their lack of growth makes it likely that their membrane traffic and cytoskeletal systems will share features that are distinct from those in cells that must be continuously expanding up to division.

## Temperature, the forgotten variable

Cycling through this morning's heavy frost deepened my gratitude at being a homeotherm, and the cells growing in my lab can also look forward to another day of consistent incubation. However, not all organisms are so lucky and indeed for much of evolutionary history organism body temperature must have varied depending on the time of day and season. Indeed, even many extant vertebrates, including some mammals, do not maintain a constant body temperature. Thus, there must be mechanisms to ensure that biological processes are robust to temperature changes, which is likely to be a particular issue for membranes where fluidity varies with temperature. Moreover, it may place a constraint on what sorts of cellular mechanisms could have emerged in evolution before we reached our homeothermic state. For instance, phase separations are coming back into fashion as a mechanism for organising membranes and the cytoplasm, but phase behaviour can be highly sensitive to temperature changes, and so to be biologically useful it must be of a type that is robust to the sorts of temperature fluctuations that our remote ancestors would have experienced on a daily basis.
